# Organic matter and water from asteroid Itokawa

**DOI:** 10.1038/s41598-021-84517-x

**Published:** 2021-03-04

**Authors:** Q. H. S. Chan, A. Stephant, I. A. Franchi, X. Zhao, R. Brunetto, Y. Kebukawa, T. Noguchi, D. Johnson, M. C. Price, K. H. Harriss, M. E. Zolensky, M. M. Grady

**Affiliations:** 1 Department of Earth Sciences, Royal Holloway University of London, Egham, TW20 0EX Surrey UK; 2 The Open University, Walton Hall, Milton Keynes, MK7 6AA UK; 3 CNRS, Institut d’Astrophysique Spatiale, Université Paris-Saclay, 91405 Orsay, France; 4 Yokohama National University, Yokohama, 240-8501 Japan; 5 Faculty of Arts and Science, Kyushu University 744, Motooka, Nishi-ku, Fukuoka, 819-0395 Japan; 6 Camborne School of Mines, University of Exeter, Penryn, Cornwall, TR10 9FE UK; 7 CAPS, School of Physical Sciences, University of Kent, Canterbury, CT2 7NH Kent UK; 8 Astromaterials Research and Exploration Science, NASA Johnson Space Center, Houston, TX 77058 USA; 9 The Natural History Museum, London, SW7 5BD UK

**Keywords:** Planetary science, Astronomy and planetary science

## Abstract

Understanding the true nature of extra-terrestrial water and organic matter that were present at the birth of our solar system, and their subsequent evolution, necessitates the study of pristine astromaterials. In this study, we have studied both the water and organic contents from a dust particle recovered from the surface of near-Earth asteroid 25143 Itokawa by the Hayabusa mission, which was the first mission that brought pristine asteroidal materials to Earth’s astromaterial collection. The organic matter is presented as both nanocrystalline graphite and disordered polyaromatic carbon with high D/H and ^15^N/^14^N ratios (δD =  + 4868 ± 2288‰; δ^15^N =  + 344 ± 20‰) signifying an explicit extra-terrestrial origin. The contrasting organic feature (graphitic and disordered) substantiates the rubble-pile asteroid model of Itokawa, and offers support for material mixing in the asteroid belt that occurred in scales from small dust infall to catastrophic impacts of large asteroidal parent bodies. Our analysis of Itokawa water indicates that the asteroid has incorporated D-poor water ice at the abundance on par with inner solar system bodies. The asteroid was metamorphosed and dehydrated on the formerly large asteroid, and was subsequently evolved via late-stage hydration, modified by D-enriched exogenous organics and water derived from a carbonaceous parent body.

## Introduction

Understanding the earliest chemical reactions involving liquid water provides crucial insights to how simple building blocks of organic compounds evolved into increasingly complex macromolecules via actions of water. Such investigation necessitates the availability of pristine samples of astromaterials—samples that have not been compromised by terrestrial contamination, and thus preserve the intrinsic states of the materials’ physical, chemical, organic and other properties^1^. Studying freshly collected, cleanly curated astromaterials returned by spacecraft reduces the ambiguity of terrestrial exposure that meteorite samples have typically experienced. The Hayabusa mission of the Japan Aerospace Exploration Agency (JAXA) is the most recently returned mission and has successfully recovered over thousands of regolith particles, with sizes ranging 10‒200 μm (typically < 50 μm in diameter), from the near-Earth S-type asteroid 25143 Itokawa in 2010^2,3^. Itokawa is considered a rubble-pile asteroid that was re-accreted from materials of a formerly large, thermally metamorphosed, collisional-disrupted precursor planetesimal^4,5^. S-type asteroids are among the most common objects in the inner asteroid belt, where the majority of Earth’s meteorites—ordinary chondrites—came from. Ordinary chondrites typically have low organic contents (insoluble carbon contents of carbonaceous chondrites = 0.051 –2.25 wt%; ordinary chondrites = 0.002 − 0.36 wt%)^6^. Hence, their organic analyses had been challenging, which is more so in the case of minute-sized returned samples of small total recovered mass.

More than 900 Itokawa particles have been separated and are curated in an ISO 6 cleanroom at the Planetary Material Sample Curation Facility of JAXA; based on mineralogy, chemistry and oxygen isotope compositions, the particles are linked directly to LL ordinary chondrites^3,7^. Organic analyses have been carried out on fewer than ten of the Itokawa particles, and all were found to contain organic matter^8–14^, which includes a group of particles (category 3 particles) composed predominantly of carbon^9–14^, despite organic carbon not generally being abundant in ordinary chondrites^6^. However, the origin of the observed organic matter has yet to be established with certainty, as its isotopic composition (δD =  − 3 to + 135‰; δ^13^C =  − 27 to + 3‰; δ^15^N =  − 4 to + 18‰) falls within a zone that overlaps with both terrestrial and some extra-terrestrial organic matter^9,14^. Parent body metamorphism in ordinary chondrites has also been shown to dramatically alter the elemental and isotopic compositions of the containing organic matter, perplexing the interpretation of the organic content of Itokawa^6^, whose rocks have experienced some degree of thermal metamorphism^3^. The water contents of the nominally anhydrous minerals (NAMs) of two Itokawa particles have recently been determined to be 698–988 parts per million (ppm), which equates to a water content of 160–510 ppm for the entire Itokawa asteroid^15^. This estimated water content is at the higher end of the range estimated for inner solar system bodies (e.g., 30–300 ppm for S-type asteroids based on remote observations of 433 Eros and 1036 Ganymede^16^; 250‒350 ppm for L and LL chondrites based on laboratory measurements^17^; 3–84 ppm in the bulk silicate Moon), except for Earth and possibly Venus which contain up to 3000 ppm of water in their bulk silicate^18^. The occurrence of organic matter and hydrogen in NAMs complicates determination of the isotopic compositions of the associated water. To address this issue, we carried out a comprehensive study to investigate the compositions of both the organic material and water contained within a single Itokawa regolith particle.

**Figure 1:**
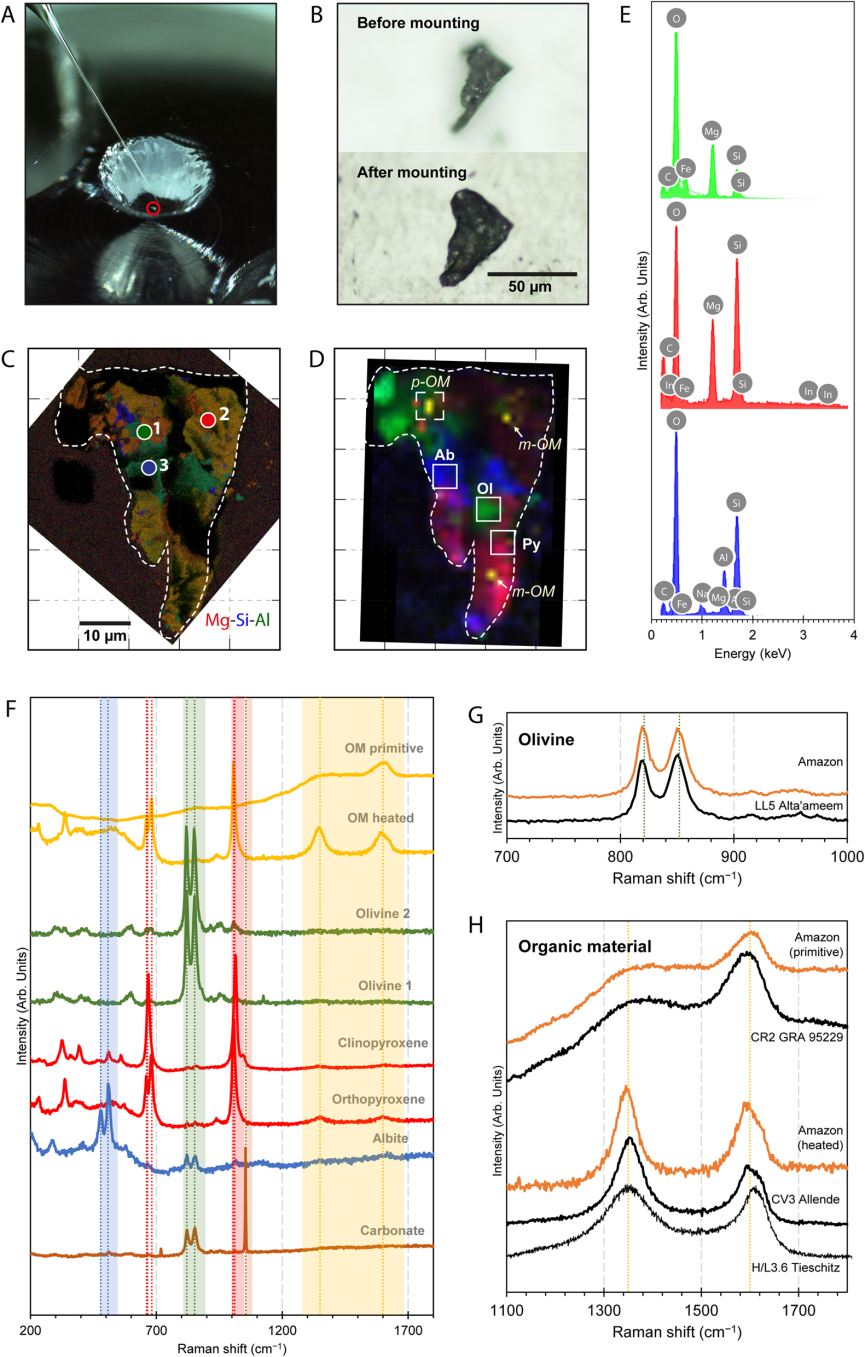
The chemical distribution and mineralogy of Amazon. (**A**) Image showing Amazon being picked up using a glass needle with platinum wires at JAXA (image provided by JAXA), (**B**) Photomicrograph taken in visible light of Amazon before and after being mounted into indium, (**C**) EDX combined Mg-Si-Al X-ray maps (Mg is red, Si is blue, Al is green) of Amazon, grids are 10 µm in size. Locations of EDX spectra in (**E**) are shown as points 1–3, and (**D**) Raman map of Amazon showing mineralogical distribution of olivine (green), plagioclase (blue), pyroxene (red) and OM (yellow). Locations of primitive OM (p-OM) and mature OM (m-OM) are marked by the italicized annotations, NanoSIMS spot analyses of albite (Ab), olivine (Ol) and pyroxene (Py) are marked as open squares, and the area of NanoSIMS imaging analysis is marked as dashed square, (**E**) EDX spectra of olivine, pyroxene and albite, locations of the points are shown in (**C**), and (**F**) selected Raman spectra of the mineral and organic components in Amazon. Peak positions of their characteristic Raman modes are shown as the dotted lines in their corresponding colors. (**G**) Selected Raman spectra of Amazon olivine compared to heated chondrite LL5 Alta-ameem. (**H**) Selected Raman spectra of Amazon organics compared to that of primitive and heated chondrites. Data of Tieschitz were from^28^.

Itokawa particle RA-QD02-0162 (approximately 30 µm across at its widest and 50 µm long) offers a unique opportunity to investigate both the water and organic contents of Itokawa (Fig. 1A,B), as it contains a wide distribution of structurally distinct organic material across the particle. The relatively large sizes (> 10 µm) of some NAM crystals also enable the study of the abundance and isotopic composition of the water (as hydroxyl group). The particle has been given the nickname “Amazon”, to recognize its unique shape resembling the South America continent preserved after soft pressing into indium (Fig. 1B). The particle has enabled direct study of the spatial distribution of preserved organics and minerals and the investigation of their relationship. Amazon is a polymineralic particle, as are a third of the returned grains^3^. The compositions of its main minerals have been determined by energy dispersive X-ray (EDX) spectroscopy (Fig. 1C,E), and Raman analysis based on the peak positions of the characteristic Raman modes^19–21^: olivine (Fo_75–85_), low-Ca pyroxene (orthopyroxene En_87_), clinopyroxene En_50_Wo_50_, albite, with a small contribution of high-temperature carbonate^22^ (Fig. 1D,F,G). An origin from an S-type asteroid can be firmly established for Amazon, as its chemical composition is comparable to LL chondrites and that reported for Itokawa samples^3,7,23^.

**Figure 2:**
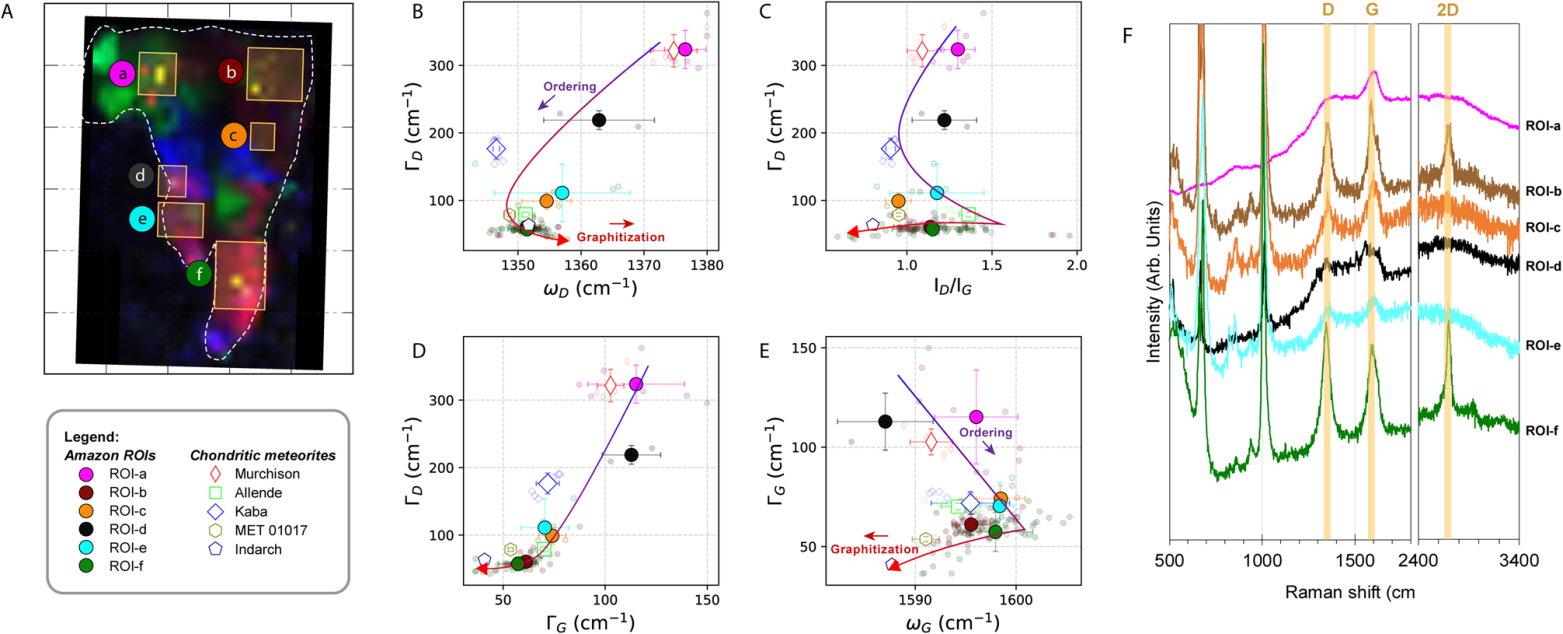
Organic composition of Amazon. (**A**) Six ROIs (a: disordered organics; b–f: heated organics) are marked by the yellow boxes. (**B**–**E**) Comparison of the Raman D and G band parameters (ω = peak center locations, Γ = full-width half-maximum, I_D_/I_G_ = peak intensity ratios between the D and G bands) of the organic material in Amazon and chondritic meteorites. The values were obtained by peak fitting to the two-peak Lorentzian model and linear baseline correction to allow comparison to literature data. Data of MET 01017 and Indarch are from^29^. Uncertainties are 1σ SD of the mean. (**F**) Selected Raman spectra of each ROI showing the first-order D and G bands, and the second-order 2D band.

Based on the observation of the Raman peak locations and widths of the first-order defect (D) band at ~ 1350 cm^−1^ and the graphite (G) band at ~ 1590 cm^−1^^24^, Amazon exhibits a significant variety of carbonaceous material (Figs. 1H, 2). It is comprised of both disordered polyaromatic organic material that shares similarity with the organics in primitive meteorites (i.e. Ivuna-type (CI), Mighei-type (CM) and Renazzo-type (CR) chondrites), as well as organic material that has been heavily graphitized (Figs. 1H, 2). The mature (“heated”) organic components occur throughout the particle as clusters of finely dispersed submicron grains and are always associated with the pyroxenes, whereas the primitive organic material occurs as a discrete grain of < 3 µm in diameter hosted within a polycrystalline mixture of olivine, pyroxene, albite and carbonate (Figs. 1, S1). The ubiquitous association of the heated organic material with pyroxenes in Amazon suggests a synthetic relationship between the two, supporting an in-situ synthetic origin of the organics on Itokawa’s pre-shattered parent body.

**Figure 3:**
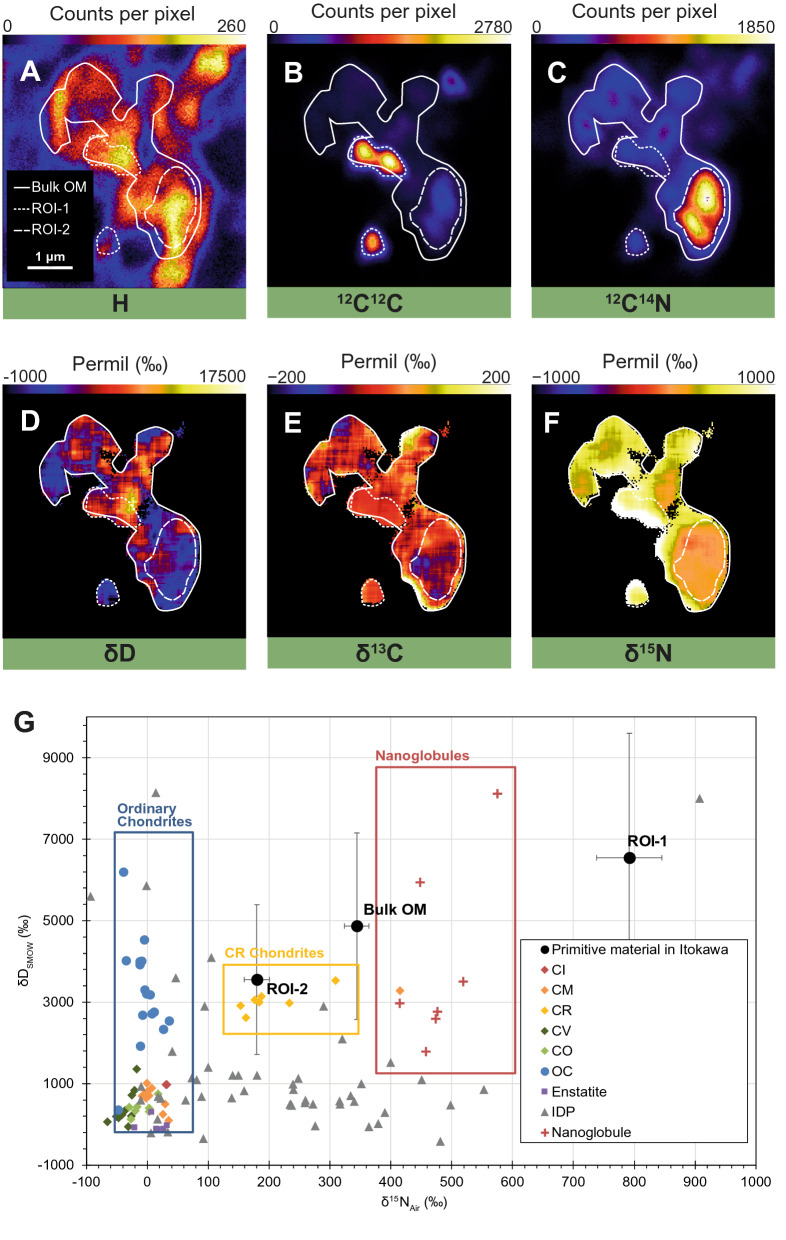
Isotopic composition of the primitive organic material in Amazon. (**A**) NanoSIMS ion image of H. (**B**) ^12^C^12^C. (**C**) ^12^C^14^N. (**D**) Isotopic images of the CN-rich region of δD, (**E**) δ^13^C and (**F**) δ^15^N. (**G**) Isotopic compositions for H versus N, for organic material in Amazon, various chondrites, interplanetary dust particles, and nanoglobules. The area marked as bulk OM was determined by CN enrichment in the ^12^C^14^N ion image, and ROI-1 and ROI-2 by enrichments in C and N in the ^12^C^12^C and ^12^C^14^N ion images, respectively. The H and N isotopic compositions of Amazon OM suggest a genetic link between the primitive organic material observed in Itokawa to CRs and IDPs. Error bars are 2 sigma errors. All data and data sources are provided in the Supplementary Material (Table S5). The NanoSIMS ion and isotopic images were processed using the IDL (L3Harris Geospatial Solutions, Inc.) based L’image software package (Larry Nittler, Carnegie Institution of Washington, http://limagesoftware.net/).

The Raman parameters of the heated organics in Amazon exhibit a prominent graphitization trend as shown by the rapidly decreasing intensity ratio between the D and G bands (I_D_/I_G_) with a continued decrease in the full width at half maximum (Γ) of the G band^25^, indicating the onset of large-scale graphitization (Fig. 2). A prominent second-order 2D overtone mode at ~ 2690 cm^−1^ alongside the narrow D and G bands shows that this heated material is present in the form of poorly-crystalline graphite^26^. The graphitization of Itokawa organics was not complete, as full-scale graphitization would have further reduced the I_D_/I_G_ ratio to close to zero. The in-plane crystallite size of graphitic domains varies inversely with I_D_/I_G_ in highly ordered organic material^24^; with this, we estimated the aromatic domain size to range from 2.2 to 7.4 nm. The organic structure of the heated material is best represented by nanocrystalline graphite (nc-G), with comparable Raman parameters to that observed for metamorphosed meteorites (e.g., ordinary chondrites L3–6 Inman, Tieschitz and New Concord^27,28^, Vigarano-type carbonaceous chondrites CV3 Allende and Meteorite Hills (MET) 01017, and enstatite chondrite EH4 Indarch^29^), suggesting peak metamorphic temperatures of at least ~ 600 °C (Figs. 2, 3). The thermal history recorded in Amazon marries well with the peak metamorphic temperature estimates for returned Itokawa regolith grains (600–800 °C)^3^. At these elevated temperatures, thermal decomposition would be at the final stage by which the mineral components would have undergone complete dehydration, contributing a large amount of localized H_2_^30^. Surface-catalyzed reactions, such as Fischer Tropsch- or Haber Bosch-type gas-grain reactions, which take place at temperatures of ~ 150 to 700 °C, could commence on mineral grain surfaces by adsorption and ab initio synthesis of simple precursor species^31^. Prolonged metamorphism would further graphitize the organic material to form nc-G. Observation of the surface of asteroid (101955) Bennu by the OSIRIS-REx mission has identified possible signature of graphitized carbon, which was suggested to indicate alteration of organic material by space weathering^32^. However, laboratory simulation experiments have also shown that organic matter can be amorphized by ion irradiation (the “opposite” of graphitization), hence whether irradiation causes amorphization or graphitization of the organic matter on an asteroid is determined by the irradiation energy (which is a function of the solar flux) and other factors such as the asteroid’s heliocentric distance, rotational velocities, density, heat conductivity. Sun-driven heating of near-Earth asteroids can penetrate a few cm into typical asteroidal surfaces, and can reach temperatures as high as 900 °C (complete dehydration of phyllosilicates) on small bodies with an orbit passing close to the sun (< 0.1 astronomical unit [AU]). Nevertheless, with a perihelion distance of 0.953 AU^4^, the surface temperature of Itokawa could only reach ~  < 120 °C^33^, which would not be sufficient to produce the strong graphitization signature we have observed for the mature OM in Amazon. Therefore, the mature OM should reflect OM ordering via metamorphism on Itokawa parent body.

The spatial distribution of the primitive organic material indicates an “exogenous” origin: it must have been incorporated into Itokawa after, and hence not graphitized by, the main episode of parent body metamorphic heating, possibly via infall of primitive carbonaceous chondrites and interplanetary dust particles (IDPs). The infall (“contamination”) of exogenous materials on asteroid surface is consistent with recent reports from laboratory experiments, computational modelling, telescopic, orbital and lander observations of Itokawa, Ryugu and Bennu, which indicated that asteroids capturing foreign materials—including hydrated asteroids—is more common than previously thought^34–36^. We have obtained the hydrogen, carbon and nitrogen isotopic compositions of the primitive organic material in Amazon to determine its origin (Fig. 3). The isotopic compositions are heterogeneous at the sub-micrometer level. The disordered organic exhibits unambiguously extra-terrestrial isotopic signatures (δD =  + 4868 ± 2288‰; δ^13^C =  − 24 ± 5‰; δ^15^N =  + 344 ± 20‰), contrasting to the typically negative isotopic values obtained for terrestrial organic matter^37^, which rules out a terrestrial/spacecraft contamination origin for Amazon. The isotopic compositions of the organic matter in chondritic meteorites vary according to their meteoritic class; a notable characteristic is the deuterium enrichment of primitive ordinary chondrites, with δD ~  + 2000 to + 6000‰^6^. The D-enrichment in organic matter in ordinary chondrites is higher than all other chondritic meteorites, except for that in CR chondrites, which falls in the same range as the ordinary chondrites. As the Hayabusa samples were recovered from the surface of Itokawa where the regolith was subjected to prolonged solar wind irradiation^38^, irradiation-driven D-enrichment could have locally equilibrated the isotopic composition between the irradiated organic solid and a deuterium plasma^39^, accounting for the systematic D-enrichment of IOM relative to solar system water reservoir. The δD and δ^13^C values of the organic material in Amazon are comparable to ordinary chondrites, however, the δ^15^N value is higher than that typically observed for ordinary chondrites (δ^15^N =  − 47 to + 36‰), and is similar to that of CRs (δ^15^N =  + 153 to + 309‰)^6^. Our data suggest a genetic link between the primitive organic material observed in Itokawa to CRs and IDPs for they share similar D, ^13^C and ^15^ N enrichments^40^ (Fig. 3G). The primitive organic matter observed in Amazon possibly represents the common component that was added to different parent bodies at various amounts, and was subsequently evolved in response to varying parent body conditions and processes.

^15^N-enrichment is a common trait of unaltered astromaterials such as IDPs and primitive meteorites. It is thought to be associated with labile material formed in cold and radiation-rich environments, as, for instance, the molecular cloud from which the solar system accumulated^41^. Such “precursor” organic matter would have been incorporated into the asteroid parent body initially as D- and ^15^N-enriched material. Subsequently, parent body metamorphism decomposed the ^15^N-rich labile components, leading to a reduction in the overall ^15^N-enrichment^42^. In contrast, ^13^C-enrichment increases with increasing metamorphic temperatures^6^ in ordinary chondrites, CRs and reduced Vigarano-like (CV) chondrites, as the organic phases can incorporate and preserve ^13^C-enriched component during graphitization under reductive thermal metamorphism (i.e. subjected to complete dehydration and reduction by carbonaceous material)^6,30,42^. The low δ^13^C value of Amazon’s primitive organic grain is comparable to that in unheated chondritic material, supporting our view of the “precursor-type” nature of the organic grain as the unaltered initial organic progenitor of astromaterials. Unfortunately, the small size of the nc-G crystallites in Amazon precluded their analysis by NanoSIMS, but isotopic characterization is still possible by techniques that offer extreme spatial and chemical resolution, such as atom probe tomography.

**Figure 4:**
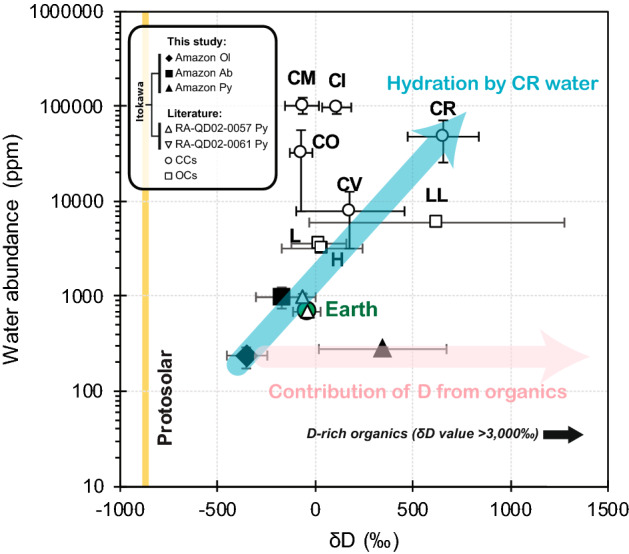
Water contents of NAMs in Amazon compared to water on bulk silicate of Earth, chondritic material and protosolar. Data indicated by closed black symbols are from this study. Error bars are 2 sigma errors. The blue arrow indicates the change in water abundance and δD value from the initial value (recorded by Itokawa olivine), towards values reported for water in Itokawa albite (this study), Itokawa pyroxene reported by Jin and Bose^15^, and Earth, by the incorporation of CR water. With a δD value of + 4868‰, organic material increases the δD value of the organic-bearing pyroxene in Amazon following the pink arrow. Literature Itokawa pyroxene data are from^15^, chondritic data are from^18^ and references therein.

We have obtained water abundances for the pyroxene and, for the first time, olivine and albite grains in an Itokawa particle (Fig. 4). While the H_2_O/δD systematic of the albite (993 ± 252 ppm (2σ) with δD value of − 177 ± 128‰) matches the values of Itokawa pyroxenes measured previously^15^, olivine and pyroxene exhibit different isotopic signatures. The water contents of olivine and pyroxene are much lower, 235 ± 60 ppm and 278 ± 14 ppm, respectively, which are comparable to that of olivines reported in ordinary chondrite Bishunpur chondrules (76–326 ppm)^43^. Organic material is ubiquitous in the pyroxenes in Amazon. In light of the prominent D-enrichment in Itokawa organics, it is imperative to take into account the presence of this organic matter when determining the overall water content of individual silicate grains within the particle. Organic material within the pyroxenes is nc-G that typically contains almost no hydrogen^44^. Despite the low contribution of the D/H from the organic component towards the overall D/H of the pyroxene, the reported water abundance and δD value should be treated as upper limits, in particular when water occurs at the ppm level, as in NAMs.

The δD value of olivine (δD =  − 354 ± 104‰) is significantly lower than that of pyroxene (δD =  + 328 ± 328‰). We attribute this discrepancy to (i) the presence of D-rich organic matter in pyroxene, and/or (ii) H_2_ dehydration/degassing of the pyroxene during thermal metamorphism^45^. Both processes would have led to an increase in the D/H ratio in the pyroxene. The olivine δD value overlaps with values estimated for water in several types of carbonaceous chondrites^46,47^, suggesting that Itokawa might have incorporated similar D-poor water ice. This observation is in agreement with the hypothesis that water of inner solar system bodies had the same initial isotopic composition^39^. Subsequently, Itokawa has undergone late-stage low-temperature hydration^48^, modified by exogenous water derived from a carbonaceous chondrite-like parent body, which is supported by the H_2_O/δD values of the late-forming albite. The incorporation of materials from an exogenous carbonaceous parent body is also in line with the observation of a 6 m black boulder on the surface of Itokawa, which was suggested to be a carbonaceous chondrite originated from an impactor of 200 − 800 m in diameter^4,5^. The catastrophic impact event(s) took place rather recently (1.3–1.4 billion years (Ga) ago)^49^, when the > 40 km in diameter parent body^50^ was cataclysmically disrupted and reassembled within the inner main asteroid belt^51^ populated by V-type, S-complex asteroids, and about a quarter of the total mass medium size (20–100 km in diameter) C-complex asteroids that are almost in equal abundance to smallest size (5–20 km in diameter) S-complex^52^. Some of these asteroids are collisional fragments of formerly larger asteroids, while some are planetesimals accreted directly from the protoplanetary disk and are typically larger than 35 km, and the latter is a likely scenario for the proto-Itokawa parent body^53^. It is also possible that the parent body has already been structurally weakened by prolonged battering by small impacts, which was subsequently catastrophically disrupted by a final low-energy impact^54^. Upon re-accretion, Itokawa spent thousands of million years residing in the main belt until being injected by Yarkovsky force into its current Earth-crossing orbit via the *ν*_6_ secular resonance^51,55^.

A single grain, Amazon, from the Itokawa asteroid, has preserved both primitive and processed organic matter within ten microns of distance. Itokawa was re-accreted from materials of a formerly large asteroid with internal heating up to 800 °C, into the present diminished-size asteroid (0.5 × 0.3 × 0.2 km^3^)^3,4^. Dehydration of silicates took place on the formerly large asteroid, which released localized H_2_ and triggered surface-catalyzed reactions to synthesize organic materials, yet the prolonged metamorphism generated immense heat that subsequently graphitized the organics. The following catastrophic impact process shattered the silicates and concluded the metamorphic regime of Itokawa. Nevertheless, continued evolution of Itokawa is evident by the infall of primitive organic material derived from CR chondrite or IDP, accounting for a complex interplay between the remnant Itokawa silicates with exogenous water and organics.

## Methods

### Sample preparation

The Itokawa particle Amazon was allocated to The Open University (OU) in the 1st International Announcement of Opportunity. Prior to distribution to OU, Amazon was removed from the collector chamber at Japan Aerospace Exploration Agency (JAXA) by JAXA curation team using glass-needle micromanipulators. At OU, Amazon was kept in the original sealed container designed by JAXA in a high-purity nitrogen (ppb levels of trace gases) atmosphere, and the container was only opened immediately prior to Amazon’s analysis in ISO Class 7 cleanroom laboratories. All tools and materials in contact with the sample were sterilized by baking in air at 500 °C for 10 h before use. Prior to high-resolution Raman mapping, Amazon was picked from the JAXA glass slide by MicroProbes tungsten micro-needles (< 1 µm tip diameter) with a micromanipulator under an optical microscope objective inside a Bassaire laminar flow hood under HEPA-filtered positive pressure (equivalent to ISO Class 4–5), and pressed flat with a spectroscopic grade sapphire window into Alfa Aesar high-purity indium (99.9999%) mounted on aluminum stubs. The same sample mounting protocol has been used to mount other small astromaterials such as interplanetary dust particles^56^.

The meteorite insoluble organic matter (IOM) samples used in Raman analysis were prepared by demineralization of the bulk meteorites with CsF-HF dissolution according to the methods described in^57^. These residues have also previously been studied by Raman spectroscopy^6,29,56,58,59^. The IOM residues were dispersed onto glass slides and directly analyzed by Raman spectroscopy.

Extra-terrestrial organic standard used for NanoSIMS imaging analysis was prepared from the Orgueil meteorite. The IOM was isolated from the Orgueil meteorites through acid maceration at room temperature in a HF/HCl mixture (2:1, v/v) as described in^60^. The Orgueil IOM residue was pressed into high-purity indium (99.999%) and gold-coated before introducing into the NanoSIMS. The same residue has also been studied previously by NanoSIMS by^60^.

Several terrestrial standards were used for NanoSIMS spot and imaging analyses. San Carlos olivine with 2 ppm of H_2_O^61^ and Bultfontein mine (South Africa) clinopyroxene (PE) with 0 ± 11.4 ppm of H_2_O as determined by elastic recoil detection analysis (ERDA)^62^ was used for background monitoring, KBH-1 orthopyroxene^61^ for instrumental mass fractionation (IMF), and 116610-18, 116610-15 and 116610-21 clinopyroxenes^63^ for H_2_O calibration. These standards were mounted in 10 mm diameter aluminum boats filled with indium following the protocol established in previous studies^64,65^. As was done previously in several studies of hydrogen in nominally anhydrous minerals (NAMS)^61,64–67^, these standards were baked overnight at 115 °C before being pressed into the indium mount. The terrestrial standards were baked in an oven at 50 °C overnight for ~ 17 h before being gold-coated and introduced into the NanoSIMS. The H_2_O concentrations and δD values of the terrestrial standards are presented in Table S1. The standards and the IDP samples were mounted on a standard geology CAMECA NanoSIMS sample holder prior to analyzing by NanoSIMS.

### Raman spectroscopy

Raman spot analysis was the first technique used to analyze Amazon when it was still on the JAXA glass slide using a Jobin–Yvon Horiba LabRam HR (800 mm) Raman microprobe at OU. The excitation source was a 514.53 nm (green) laser. More than 2500 spectra (spot and imaging modes combined) were collected for Amazon in the spectral range of 100 cm^–1^ to 4000 cm^–1^, which includes the first- (~ 1000–1800 cm^–1^) and second-order (~ 2200–3400 cm^–1^) Raman bands of carbon. The slit width and the confocal pinhole aperture were set at 150 μm and 200 μm, respectively, and a 600 grooves/mm grating was used to disperse the Raman signal, leading to a spectral resolution of approximately 3 cm^−1^. The laser beam was focused through a microscope equipped with a 100× objective (numerical aperture = 0.75). At this magnification and for the laser used, the *theoretical* minimum achievable spot size of the Raman probe was approximately 0.8 μm, and the laser power at the sample surface was ~ 150–200 μW. The exposure time for each spectrum was 20 s and three accumulations were obtained for each analytical spot to identify and discard spurious signals, such as those from cosmic rays, leading to a total acquisition time of up to 180 s. Peak position was calibrated daily against a silicon wafer prior to sample analyses and no significant shift was observed. Laser power was also checked daily prior to analyses to ensure that the laser power was consistent amongst all samples. Spectral peak identification and methods used in the present study were the same as outlined in^68^ and^59^.

High-resolution Raman mapping was also conducted after the samples were pressed into indium. Autofocus was applied prior to every analytical point in mapping mode on maximum Raman signal in the spectral region of 1580 cm^–1^ to 1600 cm^–1^ which broadly includes the first-order D and G bands. The step size was ~ 0.5 µm in both the x and y directions. The sample surface was re-examined by the optical microscope to check for any damage and we confirm that no sign of physical damage was observed.

### Curve-fitting and baseline correction

The peak position (ω) and full width half-maximum (FWHM, Γ) of each Raman band were determined by simultaneous peak fitting to the two-peak Lorentzian model and linear baseline correction so that the results can be comparable to literature data presented in^29^ who also used a two-peak Lorentzian fitting model. An example of the two-peak Lorentzian model can be found in Figs. S2 and S3. We have also fitted our data using a two-peak Lorentzian and Breit–Wigner–Fano (BWF) model^25^ can be found in Fig. S4, and the comparison between the two-peak Lorentzian and LBWF models is shown in Fig. S5. Details of the Raman peak fitting procedures and rationales are given in^59,68^. The peak parameters of the D and G bands, such as the peak center locations (usually referred to as peak position, ω), peak widths in terms of full width half-maximum (FWHM, Γ), and the peak intensity ratios between the D and G bands (I_*D*_/I_*G*_), were documented to systematically correlate with various properties of OM in meteorites. The combination of these peak parameters describes the overall size distribution of the crystalline domains and the metamorphic history of the carbonaceous host e.g.,^28,29,59,68–74^. Raman band parameters for each sample were reported as average of all selected spectra and the uncertainties are the 1σ standard error of the mean of all used spectra. Only fitted data with R^2^ values > 0.9 are shown in Fig. 2 of the main text.

### NanoSIMS imaging analysis

NanoSIMS analysis was performed by a CAMECA NanoSIMS 50L ion microprobe at OU in order to determine the H, C, and N isotopic composition of Amazon. Analyses were carried out using a Cs^+^ primary beam with a diameter of ∼1 μm and an accelerating voltage of ∼16 kV.

The NanoSIMS imaging analysis was carried out by building on the methods described in^56^ and^75^. Isotopic images were acquired in multi collection mode with electron multipliers (EMs). The analyses were conducted in two analytical set-ups: Set-up 1: (targeting C and N isotopes) ^18^O, ^12^C^12^C, ^12^C^13^C, ^12^C^14^N, ^12^C^15^N, ^28^Si^28^Si; and Set-up 2: (targeting H isotopes) ^1^H, ^2^H, ^12^C, ^13^C, ^18^O. A Cs^+^ probe with a current of 2 pA for C and N isotopes and 4 pA for H isotope was rastered over the samples a raster size of 8 × 8 μm^2^.

Prior to isotopic measurements, Amazon was pre-sputtered with a primary beam of 16 kV Cs^+^ ions and probe currents of typically approximately 10–50 pA for up to 20 min (frame size: 256 × 256 pixels; analytical area: 12 × 12 μm^2^) to remove the surface contamination and achieve approximate sputter equilibration. A frame size of 256 × 256 pixels was used for all images with an integration time of 1000 μs per pixel, leading to pixel step sizes of ~ 30 nm.

Planes of image data (C,N: 50 planes; H: 30 planes) were corrected for detector deadtime and combined, aligned and processed using the L’image software (Larry Nittler, Carnegie Institution of Washington). Data were corrected for natural isotopic and instrumental mass fractionation (IMF) relative to the isotopic values of Orguiel IOM every day before and after the analytical run of the sample (Table S2). Cold Bokkeveld IOM was used to cross-check the data. Errors are reported as two standard deviations (SD) of the mean of multiple analyses (all image planes combined for each analysis) (2σ), which have taken into consideration the error based on counting statistics, the IMF, as well as the reproducibility of standards measured during the different analytical sessions over the course of this study. An electron flood gun was used to provide charge compensation. San Carlos olivine was used for establishing background counts of electron gun contribution and NanoSIMS chamber contamination.

The spatial resolutions of primary beam of 16 kV Cs^+^ ions were determined with the L’image software to be about 122 nm for C, N, and 220 nm for H isotope measurements. A total data acquisition time of ~ 30–60 min for C, N and H isotopes. The mass resolving power (MRP) was > 9000 (according to CAMECA definition) for C/N analysis, and > 4000 for H/D analysis, sufficient to resolve all interferences from neighboring peaks.

Isotopic compositions are reported as δ values, representing the deviation of the measured isotopic ratios from reference terrestrial standards in per mil (‰), where:

1 $$\delta{\text{ R}}({{\text{per\,thousand}}})=\left(\frac{{R}_{measured}}{{R}_{reference}}-1\right)\times 1000.$$

Reference values for H, C and N isotopic ratios are determined from the reference values of the D/H ratio of the standard mean ocean water (SMOW)^76^, the ^13^C/^12^C ratio of the PeeDee Belemnite (PDB) standards^77^, and (^15^N/^14^N)_Air_^78^ (Table S2). The raw and corrected data are presented in Table S3.

Images from multiple frames were first corrected for EM dead-time set at 44 ns and then calibrated for the quasi-simultaneous arrival (QSA) effect^79^. The QSA effect has to be taken account for C in this study as the emissions of secondary ions occur at high count rates so that several secondary ions of a given isotope arrive nearly at the same time on the conversion dynode of the EM. These ions are registered as a single pulse leading to an undercount of the most abundant isotope. Values of β (the correction factor used to calculate the true isotopic ratios from the measured valued based on^79^) are phase and element specific, which had been experimentally determined for C (β = 0.6). Stage shift of typically one to three pixels (approximately 50–150 nm) during analysis was corrected during data reduction.

### NanoSIMS spot analysis

NanoSIMS spot measurements of D/H ratios and H_2_O concentrations in olivine, pyroxene and albite of Amazon were performed on the CAMECA NanoSIMS 50L ion microprobe at OU. The locations of the analytical spots were selected based on the mineralogy and surface morphology of Itokawa (Fig. S1), by ensuring that (1) the analytical spots were centered within a single mineral phase by avoiding cracks and grain boundaries, (2) the areas of the minerals of interest were larger than the sizes of the NanoSIMS sputtering and mapped surface areas discussed below, and (3) a smooth topography of the mapped areas. The H^–^, D^–^, ^13^C^–^ and ^16^O^–^ secondary ions were measured using a 16 kV Cs^+^ primary beam of 600 pA rastered over a 6 × 6 μm^2^ surface area. The electron gun was tuned to an electron current of approximately 5000 nA. ^13^C^–^ was used to monitor any potential terrestrial contamination on the sample. NanoSIMS spots are shown in Fig. 1D. Each analysis surface area was divided into 64 × 64 pixels, with a counting time of 0.132 ms per pixel. Blanking was performed, and only the 3 × 3 µm^2^ (25%) interior of the surface area was analyzed, with each measurement consisting of 2,000 cycles. Prior to the analysis, the surface was pre-sputtered for ~ 10–20 min using a primary beam current of 50 pA. Vacuum in the analytical chamber was around 3 to 3.5 × 10^–10^ Torr.

The H_2_O content of pyroxene was determined using a H^–^/^16^O^–^ vs. H_2_O calibration (Supplementary Fig. S3) based on three terrestrial clinopyroxenes (i.e., 116610-18, 116610-15 and 116610-21^63^). The slope of the calibration line is (1.62 ± 0.07) × 10^–8^. The H_2_O contents of olivine and albite were determined using a H^–^/^16^O^–^ vs. H_2_O calibration based on two orthopyroxenes (i.e., 116610-26 and 116610-29) (Table S1). The slope of the calibration line is (2.28 ± 0.42) × 10^–8^. The background for H_2_O concentrations in olivine and albite was corrected using the H^–^/^16^O^–^ ratio measured in the San Carlos olivine and the Bultfontein mine clinopyroxene (PE), corresponding to a water content of 36 ± 9 ppm (i.e. μg/g). The background for H_2_O concentrations in the pyroxene was corrected using the H^–^/^16^O^–^ ratio measured in the Bultfontein mine clinopyroxene (PE), corresponding to a water content of 16 ± 1 μg/g. This value was subtracted from each H_2_O concentration estimated in Amazon.

The instrumental mass fractionation (IMF) factor was calculated based on analyses of KBH-1, for which D/H ratio was measured by^61^ and^80^. The IMF factor was calculated to be 1.00 ± 0.04 (2SD, n = 8) for the olivine and albite session, and 1.12 ± 0.06 (2SD, n = 4) for the pyroxene session. The measured D/H ratios are expressed in terms of δD values, defined as follows in Eq. (1). The raw measured D/H ratios were corrected for IMF and the background.

The δD value measured for the San Carlos olivine was used to correct the background on the measurements of eucrite clinopyroxenes. The background corrected D/H ratio is obtained using Eq. (2):

2 $$\begin{aligned} \frac{D}{{H_{{corrected}} }} = & D/{H_{{measured}}^{{(\alpha \;corrected)}}} \times \frac{{H^{ - } /^{{16}} O_{{measured}}^{ - } }}{{H^{ - } /^{{16}} O_{{measured}}^{ - } - H^{ - } /^{{16}} O_{{background}}^{ - } }} \\ \quad &- D/H_{{background}} \times \frac{{H^{ - } /^{{16}} O_{{measured}}^{ - } }}{{H^{ - } /^{{16}} O_{{measured}}^{ - } - H^{ - } /^{{16}} O_{{background}}^{ - } }} \\ \end{aligned}$$

The data for the NAMs (including the raw H^–^/^16^O^–^ ratios and background corrected H_2_O concentrations, as well as the raw δD values and those corrected for the IMF and background) are presented in Table S4. Errors estimated for H_2_O concentrations take into account the errors from counting statistics and the background. Errors estimated for δD values take into consideration the errors based on counting statistics, as well as the errors in the IMF and on the background δD value.

### Scanning electron microscopy analysis

Initial energy dispersive X-ray (EDX) micro-analysis of Amazon has been carried out by JAXA and the data can be found at JAXA’s Data ARchive and Transmission System (DARTS) (https://darts.isas.jaxa.jp/pub/curation/hayabusa/RA-QD02-0162/). Subsequent to the Raman and NanoSIMS analyses at OU, electron images and EDX micro-analysis of Amazon were obtained with a Phenom XL Scanning Electron Microscope at OU. A low accelerating voltage was used for secondary electron (SE) imaging and EDX analysis (5 kV) to enhance the SE image resolution, analytical spatial resolution, and minimize beam damage/C contamination.

## Supplementary Information

Supplementary Information.

## Data Availability

All data needed to evaluate the conclusions in the paper are present in the paper and/or the Supplementary Materials. Additional data related to this paper may be requested from the authors.

## References

[1] McCubbin FM (2019). Advanced curation of astromaterials for planetary science. Space Sci. Rev..

[2] Yada T (2014). Hayabusa-returned sample curation in the planetary material sample curation facility of JAXA. Meteorit. Planet. Sci..

[3] Nakamura T (2011). Itokawa dust particles: A direct link between S-type asteroids and ordinary chondrites. Science.

[4] Fujiwara A (2006). The rubble-pile asteroid Itokawa as observed by Hayabusa. Science.

[5] Nagaoka H, Takasawa S, Nakamura AM, Sangen K (2014). Degree of impactor fragmentation under collision with a regolith surface—Laboratory impact experiments of rock projectiles. Meteorit. Planet. Sci..

[6] Alexander CMOD, Fogel ML, Yabuta H, Cody GD (2007). The origin and evolution of chondrites recorded in the elemental and isotopic compositions of their macromolecular organic matter. Geochim. Cosmochim. Acta.

[7] Yurimoto H (2011). Oxygen isotopic compositions of asteroidal materials returned from Itokawa by the Hayabusa mission. Science.

[8] Naraoka H (2012). Preliminary organic compound analysis of microparticles returned from Asteroid 25143 Itokawa by the Hayabusa mission. Geochem. J..

[9] Ito M (2014). H, C, and N isotopic compositions of Hayabusa category 3 organic samples. Earth Planets Space.

[10] Uesugi M (2014). Sequential analysis of carbonaceous materials in Hayabusa-returned samples for the determination of their origin. Earth Planets Space.

[11] Yabuta H (2014). X-ray absorption near edge structure spectroscopic study of Hayabusa category 3 carbonaceous particles. Earth Planets Space.

[12] Kitajima F (2015). A micro-Raman and infrared study of several Hayabusa category 3 (organic) particles. Earth Planets Space.

[13] Naraoka H (2015). ToF-SIMS analysis of carbonaceous particles in the sample catcher of the Hayabusa spacecraft. Earth Planets Space.

[14] Uesugi M (2019). Further characterization of carbonaceous materials in Hayabusa-returned samples to understand their origin. Meteorit. Planet. Sci..

[15] Jin Z, Bose M (2019). New clues to ancient water on Itokawa. Sci. Adv..

[16] Rivkin AS, Howell ES, Emery JP, Sunshine J (2018). Evidence for OH or H2O on the surface of 433 Eros and 1036 Ganymed. Icarus.

[17] Jarosewich E (1990). Chemical analyses of meteorites: A compilation of stony and iron meteorite analyses. Meteoritics.

[18] McCubbin FM, Barnes JJ (2019). Origin and abundances of H_2_O in the terrestrial planets, moon, and asteroids. Earth Planet. Sci. Lett..

[19] Kuebler KE, Jolliff BL, Wang A, Haskin LA (2006). Extracting olivine (Fo–Fa) compositions from Raman spectral peak positions. Geochim. Cosmochim. Acta.

[20] Huang E, Chen CH, Huang T, Lin EH, Xu J-A (2000). Raman spectroscopic characteristics of Mg-Fe-Ca pyroxenes. Am. Miner..

[21] Freeman JJ, Wang A, Kuebler KE, Jolliff BL, Haskin LA (2008). Characterization of natural feldspars by Raman spectroscopy for future planetary exploration. Can. Mineral..

[22] Gillet P, Biellmann C, Reynard B, McMillan P (1993). Raman spectroscopic studies of carbonates part I: High-pressure and high-temperature behaviour of calcite, magnesite, dolomite and aragonite. Phys. Chem. Miner..

[23] Bonal L (2015). Visible-IR and Raman microspectroscopic investigation of three Itokawa particles collected by Hayabusa: Mineralogy and degree of space weathering based on nondestructive analyses. Meteorit. Planet. Sci..

[24] Tuinstra F, Koenig JL (1970). Raman spectrum of graphite. J. Chem. Phys..

[25] Ferrari AC, Robertson J (2000). Interpretation of Raman spectra of disordered and amorphous carbon. Phys. Rev. B.

[26] Fries M, Steele A (2008). Graphite whiskers in CV3 meteorites. Science.

[27] Makjanic J, Vis RD, Hovenier JW, Heymann D (1993). Carbon in the matrices of ordinary chondrites. Meteoritics.

[28] Quirico E, Raynal P-I, Bourot-Denise M (2003). Metamorphic grade of organic matter in six unequilibrated ordinary chondrites. Meteorit. Planet. Sci..

[29] Busemann H, Alexander MOD, Nittler LR (2007). Characterization of insoluble organic matter in primitive meteorites by microRaman spectroscopy. Meteorit. Planet. Sci..

[30] Nakamura T (2005). Post-hydration thermal metamorphism of carbonaceous chondrites. J. Mineral. Petrol. Sci..

[31] Pizzarello S (2012). Catalytic syntheses of amino acids and their significance for nebular and planetary chemistry. Meteorit. Planet. Sci..

[32] DellaGiustina DN (2020). Variations in color and reflectance on the surface of asteroid (101955) Bennu. Science.

[33] Delbo M, Mueller M, Emery JP, Rozitis B, Capria MT (2015). Asteroids IV.

[34] Daly RT, Schultz PH (2015). Predictions for impactor contamination on Ceres based on hypervelocity impact experiments. Geophys. Res. Lett..

[35] Tatsumi E (2020). Collisional history of Ryugu’s parent body from bright surface boulders. Nat. Astron..

[36] Avdellidou C, Delbo M, Fienga A (2018). Exogenous origin of hydration on asteroid (16) Psyche: The role of hydrated asteroid families. Month. Not. R. Astron. Soc..

[37] Pizzarello S (2006). The chemistry of life's origin: A carbonaceous meteorite perspective. Acc. Chem. Res..

[38] Matsumoto T, Harries D, Langenhorst F, Miyake A, Noguchi T (2020). Iron whiskers on asteroid Itokawa indicate sulfide destruction by space weathering. Nat. Commun..

[39] Laurent B (2015). The deuterium/hydrogen distribution in chondritic organic matter attests to early ionizing irradiation. Nat. Commun..

[40] Busemann H (2006). Interstellar chemistry recorded in organic matter from primitive meteorites. Science.

[41] Füri E, Marty B (2015). Nitrogen isotope variations in the solar system. Nat. Geosci..

[42] Sephton MA (2003). Investigating the variations in carbon and nitrogen isotopes in carbonaceous chondrites. Geochim. Cosmochim. Acta.

[43] Stephant A, Remusat L, Robert F (2017). Water in type I chondrules of Paris CM chondrite. Geochim. Cosmochim. Acta.

[44] Casiraghi C, Ferrari AC, Robertson J (2005). Raman spectroscopy of hydrogenated amorphous carbons. Phys. Rev. B.

[45] Hauri EH, Gaetani GA, Green TH (2006). Partitioning of water during melting of the Earth's upper mantle at H_2_O-undersaturated conditions. Earth Planet. Sci. Lett..

[46] Alexander CMOD (2012). The provenances of asteroids, and their contributions to the volatile inventories of the terrestrial planets. Science.

[47] Piani L, Yurimoto H, Remusat L (2018). A dual origin for water in carbonaceous asteroids revealed by CM chondrites. Nat. Astron..

[48] Kyser TK, O'Neil JR (1984). Hydrogen isotope systematics of submarine basalts. Geochim. Cosmochim. Acta.

[49] Park J (2015). ^40^Ar/^39^Ar age of material returned from asteroid 25143 Itokawa. Meteorit. Planet. Sci..

[50] Wakita S, Nakamura T, Ikeda T, Yurimoto H (2014). Thermal modeling for a parent body of Itokawa. Meteorit. Planet. Sci..

[51] Michel P, Yoshikawa M (2006). Dynamical origin of the asteroid (25143) Itokawa: The target of the sample-return Hayabusa space mission. A&A.

[52] DeMeo FE, Alexander CMOD, Walsh KJ, Chapman CR, Binzel RP (2015). Asteroids IV.

[53] Delbo M, Walsh K, Bolin B, Avdellidou C, Morbidelli A (2017). Identification of a primordial asteroid family constrains the original planetesimal population. Science.

[54] Jourdan F (2017). Collisional history of asteroid Itokawa. Geology.

[55] Terada K (2018). Thermal and impact histories of 25143 Itokawa recorded in Hayabusa particles. Sci. Rep..

[56] Chan QHS (2020). Organics preserved in anhydrous interplanetary dust particles: Pristine or not?. Meteorit. Planet. Sci..

[57] Cody GD, Alexander CMOD (2005). NMR studies of chemical structural variation of insoluble organic matter from different carbonaceous chondrite groups. Geochim. Cosmochim. Acta.

[58] Starkey NA, Franchi IA, Alexander CMOD (2013). A Raman spectroscopic study of organic matter in interplanetary dust particles and meteorites using multiple wavelength laser excitation. Meteorit. Planet. Sci..

[59] Chan QHS (2019). Heating experiments of the Tagish Lake meteorite: Investigation of the effects of short-term heating on chondritic organics. Meteorit. Planet. Sci..

[60] Tartèse R, Chaussidon M, Gurenko A, Delarue F, Robert F (2018). Insights into the origin of carbonaceous chondrite organics from their triple oxygen isotope composition. Proc. Natl. Acad. Sci..

[61] Koga K, Hauri E, Hirschmann M, Bell D (2003). Hydrogen concentration analyses using SIMS and FTIR: Comparison and calibration for nominally anhydrous minerals. Geochem. Geophys. Geosyst..

[62] Barnes, J. J. *Water in the Moon: A Geochemical Approach*. PhD thesis, The Open University (2014).

[63] Kumamoto KM, Warren JM, Hauri EH (2017). New SIMS reference materials for measuring water in upper mantle minerals. Am. Miner..

[64] Aubaud C (2007). Intercalibration of FTIR and SIMS for hydrogen measurements in glasses and nominally anhydrous minerals. Am. Mineral..

[65] Mosenfelder JL (2011). Analysis of hydrogen in olivine by SIMS: Evaluation of standards and protocol. Am. Mineral..

[66] Hauri E (2002). SIMS analysis of volatiles in silicate glasses: 1 Calibration, matrix effects and comparisons with FTIR. Chem. Geol..

[67] Tenner TJ, Hirschmann MM, Withers AC, Hervig RL (2009). Hydrogen partitioning between nominally anhydrous upper mantle minerals and melt between 3 and 5 GPa and applications to hydrous peridotite partial melting. Chem. Geol..

[68] Chan QHS, Zolensky ME, Bodnar RJ, Farley C, Cheung JCH (2017). Investigation of organo-carbonate associations in carbonaceous chondrites by Raman spectroscopy. Geochim. Cosmochim. Acta.

[69] Beyssac O, Goffé B, Chopin C, Rouzaud JN (2002). Raman spectra of carbonaceous material in metasediments: A new geothermometer. J. Metamorph. Geol..

[70] Bonal L, Bourot-Denise M, Quirico E, Montagnac G, Lewin E (2007). Organic matter and metamorphic history of CO chondrites. Geochim. Cosmochim. Acta.

[71] Bonal L, Quirico E, Bourot-Denise M, Montagnac G (2006). Determination of the petrologic type of CV3 chondrites by Raman spectroscopy of included organic matter. Geochim. Cosmochim. Acta.

[72] Aoya M (2010). Extending the applicability of the Raman carbonaceous-material geothermometer using data from contact metamorphic rocks. J. Metamorph. Geol..

[73] Kouketsu Y (2014). A new approach to develop the Raman carbonaceous material geothermometer for low-grade metamorphism using peak width. Island Arc..

[74] Homma Y, Kouketsu Y, Kagi H, Mikouchi T, Yabuta H (2015). Raman spectroscopic thermometry of carbonaceous material in chondrites: Four-band fitting analysis and expansion of lower temperature limit. J. Mineral. Petrol. Sci..

[75] Starkey NA, Franchi IA (2013). Insight into the silicate and organic reservoirs of the comet forming region. Geochim. Cosmochim. Acta.

[76] Hagemann R, Nief G, Roth E (1970). Absolute isotopic scale for deuterium analysis of natural waters. Absolute D/H ratio for SMOW. Tellus.

[77] Craig H (1953). The geochemistry of the stable carbon isotopes. Geochim. Cosmochim. Acta.

[78] Mariotti A (1983). Atmospheric nitrogen is a reliable standard for natural ^15^N abundance measurements. Nature.

[79] Slodzian G, Hillion F, Stadermann FJ, Zinner E (2004). QSA influences on isotopic ratio measurements. Appl. Surf. Sci..

[80] Bell DR, Ihinger PD (2000). The isotopic composition of hydrogen in nominally anhydrous mantle minerals. Geochim. Cosmochim. Acta.

