# Publisher Correction: Organic matter and water from asteroid Itokawa

**DOI:** 10.1038/s41598-021-96583-2

**Published:** 2021-08-17

**Authors:** Q. H. S. Chan, A. Stephant, I. A. Franchi, X. Zhao, R. Brunetto, Y. Kebukawa, T. Noguchi, D. Johnson, M. C. Price, K. H. Harriss, M. E. Zolensky, M. M. Grady

**Affiliations:** 1 Department of Earth Sciences, Royal Holloway University of London, Egham, TW20 0EX Surrey UK; 2 The Open University, Walton Hall, Milton Keynes, MK7 6AA UK; 3 CNRS, Institut d’Astrophysique Spatiale, Université Paris-Saclay, 91405 Orsay, France; 4 Yokohama National University, Yokohama, 240‑8501 Japan; 5 Faculty of Arts and Science, Kyushu University 744, Motooka, Nishi‑ku, Fukuoka, 819‑0395 Japan; 6 Camborne School of Mines, University of Exeter, Penryn, Cornwall, TR10 9FE UK; 7 CAPS, School of Physical Sciences, University of Kent, Canterbury, CT2 7NH Kent UK; 8 Astromaterials Research and Exploration Science, NASA Johnson Space Center, Houston, TX 77058 USA; 9 The Natural History Museum, London, SW7 5BD UK

Correction to: *Scientific Reports* 10.1038/s41598-021-84517-x, published online 04 March 2021

The original version of this Article contained an error in Reference 32, which was incorrectly given as:

DellaGiustina, D. N. *et al.* Exogenic basalt on asteroid (101955) Bennu. *Nat. Astron.* https://doi.org/10.1038/s41550-020-1195-z (2020).

The correct reference is listed below:

DellaGiustina, D. N. *et al.* Variations in color and reflectance on the surface of asteroid (101955) Bennu. *Science* **370**, abc3660. https://doi.org/10.1126/science.abc3660 (2020).

The original Article has been corrected.

