# Supplementary material for: Organic matter and water from asteroid Itokawa
Source: Sci Rep. 2021 Mar 4;11:5125. doi: 10.1038/s41598-021-84517-x (PMC7933418; doi:10.1038/s41598-021-84517-x)
Supplement: Supplementary file 1 — Supplementary Information. [file 41598_2021_84517_MOESM1_ESM.pdf]

Supplementary Materials for

Organic Matter and Water from Asteroid Itokawa

Q. H S. Chan[1,2]*, A. Stephant[2], I. A. Franchi[2], X. Zhao[2], R. Brunetto[3], Y. Kebukawa[4], T. Noguchi[5], D. Johnson[2,6], M. C. Price[7], K. H. Harriss[7], M. E. Zolensky[8], M. M. Grady[2,9]

*Corresponding author: Queenie.Chan@rhul.ac.uk

This PDF file includes:

Figs. S1 to S5
Tables S1 to S5
References S1 to S5

A. Supplementary Figures

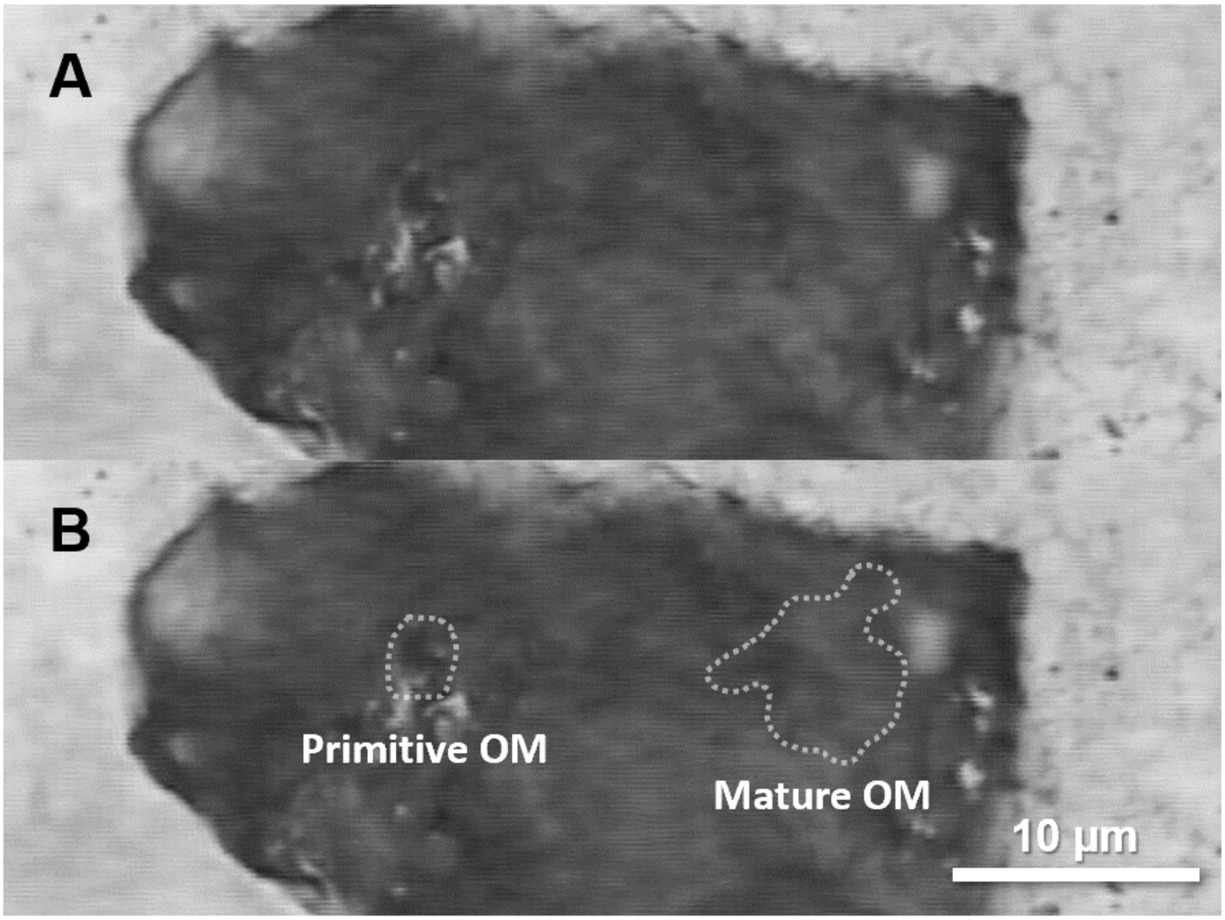

Fig. S1

Photomicrograph taken in visible light of Amazon showing the locations and occurrences of the primitive and mature OM with and without illustration.

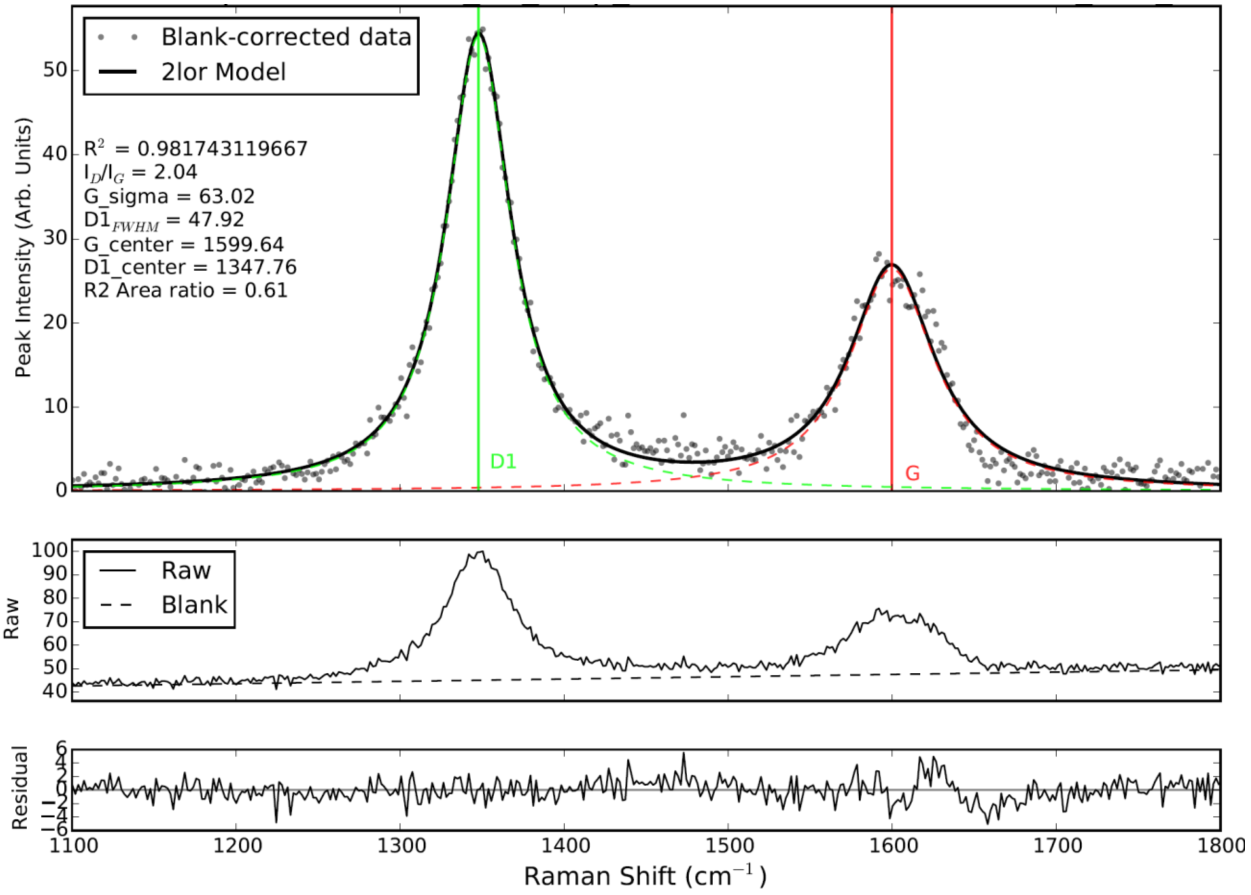

Fig. S2

Example of a two-Lorentzian fit performed on the Raman spectrum of the mature organic material in Amazon. The middle plot shows that the fluorescence background has been subtracted from the raw spectrum assuming a linear baseline. Minimal misfit was observed as displayed by the residual spectrum.

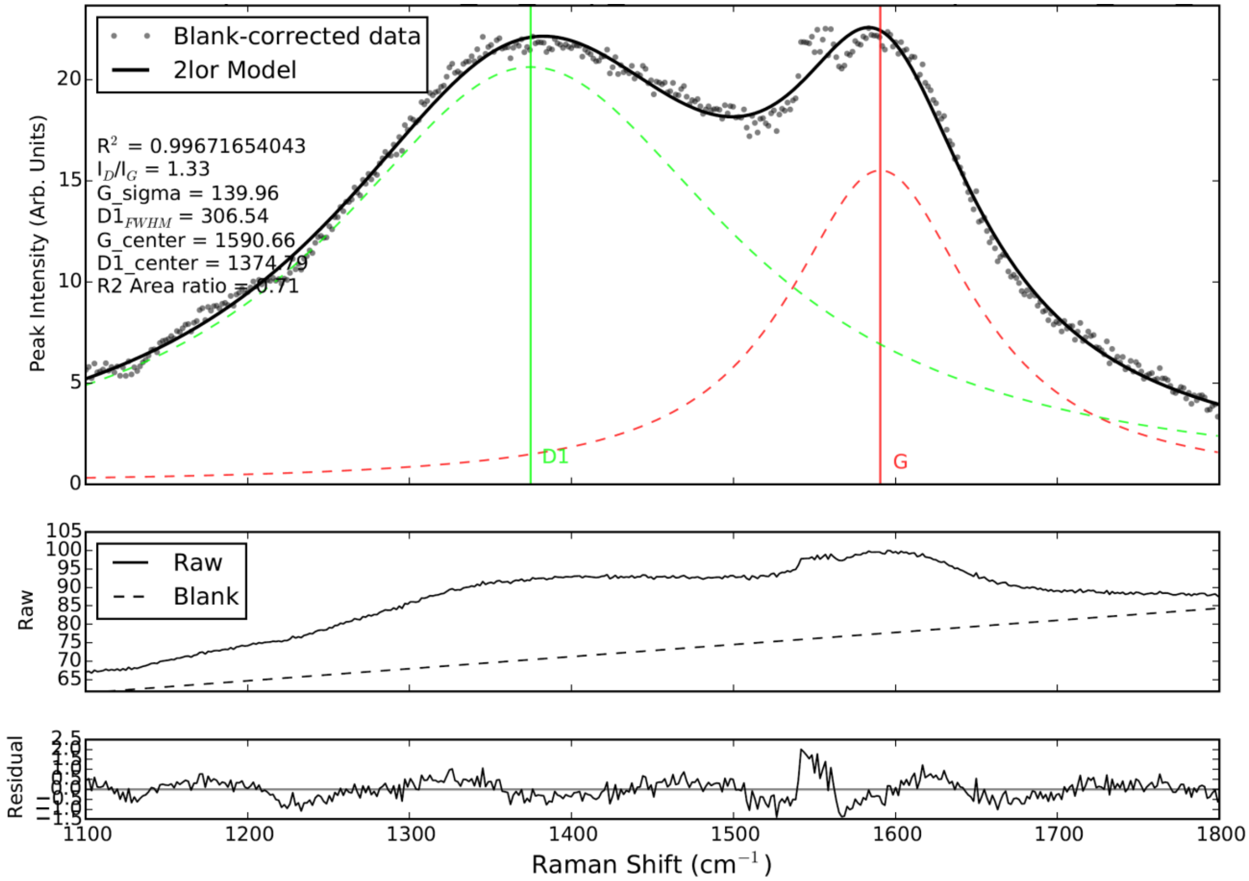

Fig. S3

Example of a two-Lorentzian fit performed on the Raman spectrum of the primitive organic material in Amazon. The middle plot shows that the fluorescence background has been subtracted from the raw spectrum assuming a linear baseline. Minimal misfit was observed as displayed by the residual spectrum.

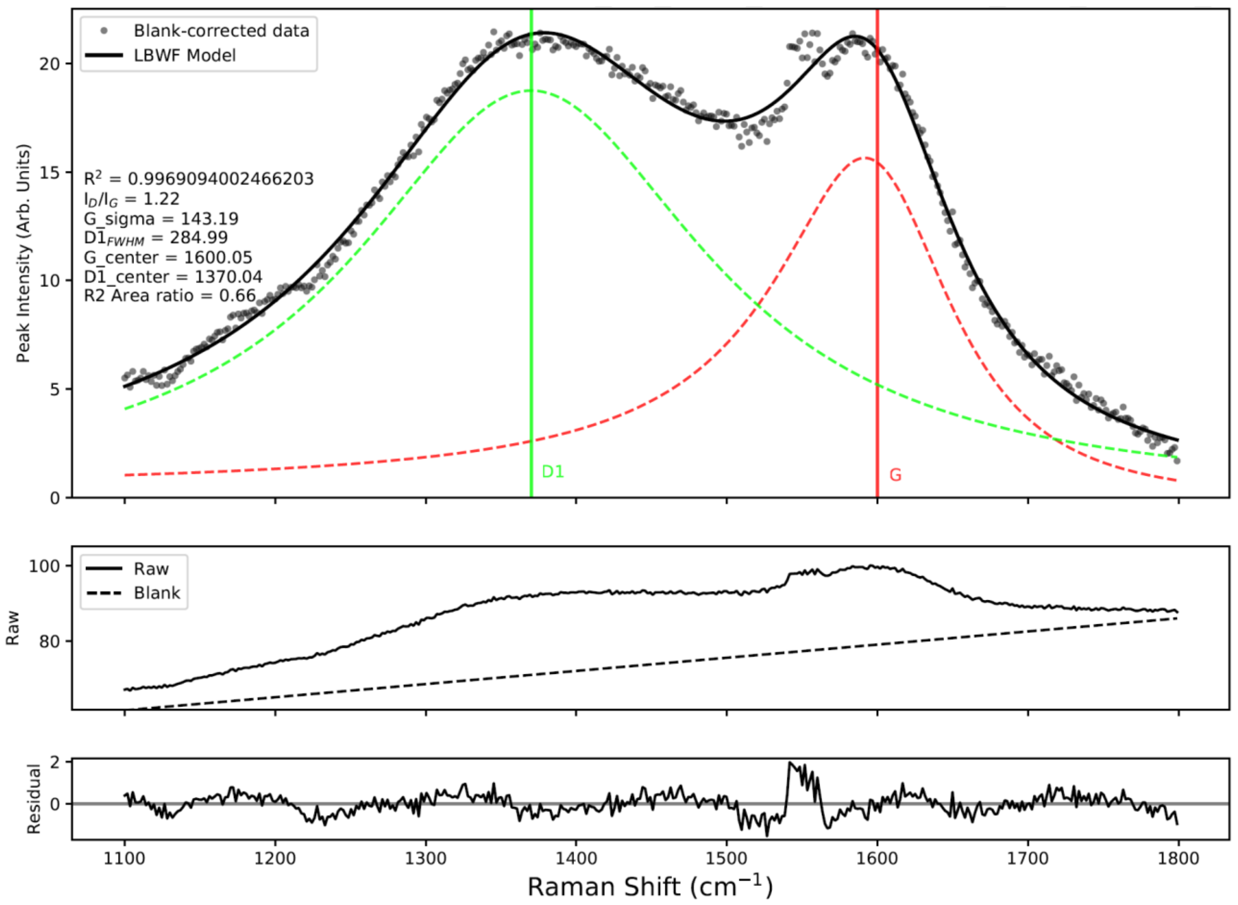

Fig. S4

Example of a two-peak Lorentzian and Breit–Wigner–Fano (LBWF) model fitting performed on the Raman spectrum of the primitive organic material in Amazon. The middle plot shows that the fluorescence background has been subtracted from the raw spectrum assuming a linear baseline. Minimal misfit was observed as displayed by the residual spectrum.

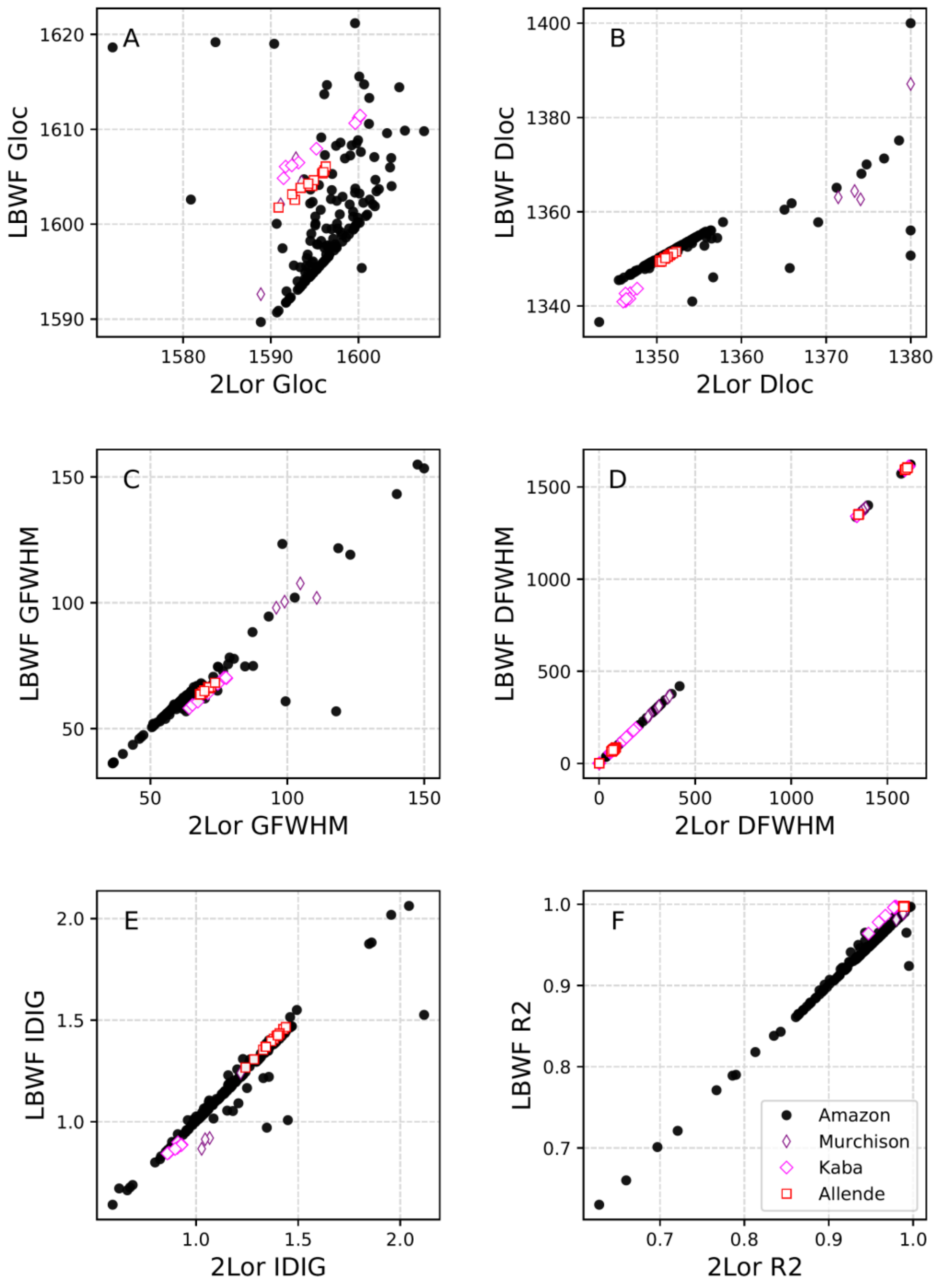

Fig. S5

Comparison between the Raman band parameters **(A)** G band location, **(B)** D band location, **(C)** G band full width at half maximum (FWHM), **(D)** D band FWHM, **(E)** intensity ratio between the D and G bands, **(F)** R^2 values of the results, obtained from peak-fitting using 2-peak Lorentzian and 2-peak Lorentzian and Breit–Wigner–Fano (LBWF) models.

B. Supplementary Tables

Table S1

The H_2O contents (ppm) and δD values (‰) of standards used for SIMS calibration of water content and for determining the instrumental mass fractionation (IMF) factor.

**Standard**	**Type**	**H_2O (ppm)**	**δD (‰)**	**References**
San Carlos	Olivine	2	-	[61]
PE [BM.1998.P2 (1780)]	Pyroxene	0 ± 11.4		[62]
KBH-1	Pyroxene	186	−113 ± 4	[61]
116610-15	Pyroxene	441 ± 31	-	[63]
116610-18	Pyroxene	199 ± 13	-	[63]
116610-21	Pyroxene	354 ± 28	-	[63]
116610-26	Pyroxene	237 ± 19	-	[63]
116610-29	Pyroxene	62 ± 4	-	[63]

Table S2

Isotopic compositions of Orgueil IOM standard used for NanoSIMS imaging analysis, and the standard ratios used in the calculation of the isotopic value of Amazon.

**Isotopic compositions (‰)**	**δD_{SMOW}**	**$\delta^{13}C_{PDB}$**		**$\delta^{15}N_{Air}$**
Orgueil IOM	+972 ± 2	–17.05 ± 0.04		+30.7 ± 0.2
Reference	[6]	[6]		[6]

	**D/H_{SMOW}**	**$^{13}C/^{12}C_{PDB}$**	**$^{12}C^{13}C/^{12}C^{12}C$**	**$^{15}N/^{14}N_{Air}$**
Standard ratios	0.00015576	0.0112372	0.0224744	0.003676
Reference	[76]	[77]		[78]

IOM: Insoluble organic matter; SMOW: Standard Mean Ocean Water; PDB: Pee Dee Belemnite; Air: atmospheric

Table S3

The raw and IMF-corrected C, N and H isotopic ratios and the delta values.

	**Bulk OM**			**ROI-1**			**ROI-2**		
ROIAREA (μm^2)	6.6			0.9			1.4		
ROIDIAM (μm^2)	2.9			1.1			1.3		
**Raw data:**									
$^{12}C^{13}C/^{12}C^{12}C$	0.01095	±	0.00006	0.01103	±	0.00007	0.01069	±	0.00012
$^{12}C^{15}N/^{12}C^{14}N$	0.00488	±	0.00004	0.00650	±	0.00018	0.00428	±	0.00005
D/H	0.00045	±	0.00002	0.00057	±	0.00007	0.00035	±	0.00004
**IMF-corrected ratios:**									
$^{12}C^{13}C/^{12}C^{12}C$	0.01097	±	0.00006	0.01105	±	0.00007	0.01071	±	0.00012
$^{12}C^{15}N/^{12}C^{14}N$	0.00494	±	0.00007	0.00659	±	0.00020	0.00434	±	0.00008
D/H	0.00091	±	0.00036	0.00118	±	0.00048	0.00071	±	0.00029
**IMF-corrected delta values (‰):**									
$\delta^{13}C$	-23.97	±	5.22	-16.76	±	6.39	-46.54	±	11.02
$\delta^{15}N$	344.32	±	20.05	791.88	±	53.44	179.96	±	20.94
δD	4868.31	±	2288.13	6542.67	±	3056.06	3554.41	±	1837.33

Table S4

The raw $H^-/^{16}O^-$ ratios and background corrected H_2O concentrations, and the raw δD values and those corrected for the IMF and background.

NAMs	D/H	D/H Poisson	$H/^{16}O$	$H/^{16}O$ Poisson	H_2O (ppm)	2SD	δD (‰)	2SD
Olivine	0.000105	5.7	0.00000617	0.065	235	60	-354	104
Albite	0.000128	5.38	0.00002355	0.0589	993	252	-177	128
Pyroxene	0.000223	6.11	0.000477	0.0912	278	14	348	328

Table S5

The C, N and H isotopic compositions for organic material in Amazon, various chondrites, interplanetary dust particles, and nanoglobules presented in Fig. 3 in the main text, and their corresponding data sources.

**Samples**	**Samples**	**$\delta^{13}C_{PDB}$ (‰)**	**$\delta^{15}N_{Air}$ (‰)**	**δD_{SMOW} (‰)**	**Reference**
Bulk OM	Amazon	-23.97	344.32	4868	This study
ROI-1	Amazon	-16.76	791.88	6543	This study
ROI-2	Amazon	-46.54	179.96	3554	This study
Orgueil	CI	-17.05	30.70	972	[6]
Ivuna	CI	-16.97	31.90	978	[6]
Bells	CM	-34.20	415.30	3283	[6]
Kivasvaara	CM	-17.13	2.70	707	[6]
Murchison	CM	-18.91	-1.00	777	[6]
Mighei	CM	-18.33	0.50	803	[6]
Murray	CM	-18.05	7.50	893	[6]
DOM03183	CM	-17.45	-1.90	639	[6]
Cold Bokk	CM	-18.63	-2.00	734	[6]
ALH83100	CM	-17.86	-6.70	723	[6]
MET01070	CM	-18.17	-8.50	693	[6]
LEW85311	CM	-15.62	-0.50	1008	[6]
Y793321	CM	-8.39	35.10	95	[6]
PCA91008	CM	-11.38	26.00	244	[6]
Y86720	CM	-19.86	29.50	496	[6]
MET00426	CR	-20.32	176.80	3054	[6]
QUE99177	CR	-20.83	187.40	3139	[6]
EET92042	CR	-22.19	184.10	3002	[6]
GRA95229	CR	-21.61	153.40	2909	[6]
Al Rais	CR	-24.20	161.80	2619	[6]
GRO95577	CR	-26.58	233.20	2973	[6]
LEW85332	CR	-23.70	309.00	3527	[6]
Kaba	CV	-15.44	-26.20	209	[6]
Mokoia	CV	-15.04	-36.30	341	[6]
MET00430	CV	-18.35	-65.60	62	[6]
Bali	CV	-18.00	-47.50	160	[6]
Allende	CV	-17.03	-51.20	194	[6]
ALH84028	CV	-15.89	-41.40	239	[6]
Leoville	CV	-13.09	-17.50	1361	[6]
Vigarano	CV	-12.05	-21.30	839	[6]
Eframovka	CV	-13.11	-25.70	714	[6]
MET01017	CV	-6.79	-31.40	-57	[6]
ALH77307	CO	-8.29	3.00	314	[6]
Colony	CO	-8.68	17.70	749	[6]
Y81020	CO	-8.17	3.30	402	[6]
Kainsaz	CO	-13.92	-16.40	435	[6]
Y791717	CO	-7.13	-19.40	340	[6]
ALH77003	CO	-7.72	-27.10	132	[6]
ALH83108	CO	-4.47	-29.70	417	[6]

Semarkona	OC	-23.67	26.90	2322	6
QUE97008	OC	-20.35	-0.70	3199	6
MET00526	OC	-15.94	-4.20	4526	6
MET00452	OC	-16.05	-7.20	2678	6
Bishunpur	OC	-16.65	12.10	2749	6
LEW86018	OC	-15.88	36.30	2531	6
GRO95502a	OC	-11.90	-3.70	3297	6
GRO95504a	OC	-10.42	5.30	3174	6
GRO95505a	OC	-15.81	-34.40	4009	6
MET96503b	OC	-13.60	-11.30	3984	6
MET96515b	OC	-13.36	-8.70	3997	6
Krymka	OC	-17.38	-10.70	1917	6
WSG95300	OC	-12.00	-38.50	6181	6
ALH83010	OC	-14.81	-46.90	346	6
Chainpur	OC	-16.86	8.20	2708	6
MET00506	OC	-13.13	-11.30	3908	6
GRO95517	EH	-7.75	27.40	-115	6
PCA91238	EH	-7.14		-160	6
ALH77295	EH	-7.36	32.50	-23	6
EET87746	EH	-7.16	6.90	299	6
Indarch	EH	-8.73	-20.90	-82	6
PCA91020	EL	-8.21	17.80	-119	6
MAC02837	EL	-7.06	14.40	-109	6
G 15-1	Nanoglobule	-47.00	474.00	2589	S1
G 15-2	Nanoglobule	-18.00	415.00	2977	S1
G 15-3	Nanoglobule	-33.00	477.00	2767	S1
G 8-1	Nanoglobule	17.00	1007.00	3097	S1
G 8-2	Nanoglobule	-42.00	575.00	8113	S1
G 8-3	Nanoglobule	-26.00	448.00	5940	S1
G 5-1	Nanoglobule	-47.00	519.00	3509	S1
G 5-2	Nanoglobule	-35.00	458.00	1789	S1
Lumley 1a	IDP	-22.00	334.00	702	S2
Lumley 1b	IDP	17.00	498.00	480	S2
Lumley1c	IDP	-14.00	259.00	531	S2
Lumley1d	IDP	-25.00	316.00	575	S2
Lumley1e	IDP	-4.00	553.00	861	S2
Lumley1f	IDP	-46.00	236.00	495	S2
Balmoral1 Spot 1	IDP	-33.00	248.00	1128	S2
Balmoral1 Spot 2	IDP	-26.00	272.00	498	S2
Balmoral1 ROI 2	IDP	-44.00	317.00	502	S2
Balmoral1 ROI 3	IDP	-42.00	352.00	1000	S2
Midford1bulk	IDP	-17.00	14.20	8146	75
Midford2bulk	IDP	-8.00	-1.00	5862	75
Bramber1bulk	IDP	-3.00	-167.00	1166	75
Bramber2bulk	IDP	-12.00	22.00	642	75
Arundel1bulk	IDP	-7.00	-190.00	498	75
Arundel3abulk	IDP	-10.00	239.00	988	75
Pizarrob	IDP	7.00	390.00	297	S3
Cortes	IDP	15.00	340.00	560	S3

Magellan	IDP	14.00	260.00	593	S3
Columbus	IDP	23.00	400.00	1525	S3
Polo	IDP	12.00	240.00	862	S3
Hudson	IDP	22.00	89.00	678	S3
Raleigh	IDP	16.00	41.00	1787	S3
Hawkins	IDP	21.00	63.00	599	S3
Drake	IDP	-1.00	73.00	1141	S3
Frobisher	IDP	23.00	139.00	651	S3
L2005 A2a 31	IDP	-4.00	-10.70	600	S4
L2005 F 31	IDP	-26.00	153.40	20000	S4
L2005 4 31	IDP	-49.00	161.80	24800	S4
L2005 3 31	IDP	-31.00	907.25	8000	S4
L2005 A3 31	IDP	-1.00	481.57	-420	S4
L2008 B2a 4	IDP	-45.00	450.97	1100	S4
L2008 310x 5	IDP	-4.00	873.07	71	S4
L2008 310d 5	IDP	-11.00	1281.99	66	S4
L2008 310e 5	IDP	33.00	379.05	17	S4
L2008 310g 5	IDP	-7.00	275.46	-35	S4
L2008 310L 5	IDP	-23.00	364.30	-55	S4
L2008 19a 5	IDP	-16.00	681.60	664	S4
L2008 110 5	IDP	-18.00	159.02	822	S4
L2009 D9 10	IDP	-52.00	80.82	12000	S4
L2009 D10 10	IDP	-42.00	80.82	1100	S4
L2009 D12 13	IDP	-120.00	105.00	4100	S4
L2009 D1 3	IDP	-52.00	320.00	2100	S4
L2009 D6 7	IDP	-43.00	140.00	1200	S4
L2009 D7 8	IDP	-1.00	150.00	1200	S4
L2011 A1 6	IDP	-38.00	180.00	1200	S4
L2011 A2 6	IDP	-14.00	100.00	1400	S4
L2011 A3 7	IDP	-53.00	-93.00	5600	S4
L2011 A4 7	IDP	-55.00	-10.00	930	S4
L2011 A5 11	IDP	-27.00	94.00	2900	S4
L2011 A6 11	IDP	33.00	290.00	2900	S4
L2011 A7 15	IDP	-76.00	17.00	130	S4
L2011 A8 15	IDP	-67.00	47.00	3600	S4
L2011 A10 22	IDP	-41.00	18.00	680	S4
L2011 4 5	IDP	-24.00	92.00	-350	S4
L2011 4	IDP	-30.00	234.00	490	S5
Chocha B 8C	IDP	-30.00	237.00	470	S5
Chuki 5-4D	IDP	-11.00	6.00	-210	S5
Chuki 5-4D	IDP	-8.00	34.00	-185	S5
Chuki 5-4D-1	IDP	2.00	915.00	960	S5

C. Supplementary References

S1 Nakamura-Messenger, K., Messenger, S., Keller, L. P., Clemett, S. J. & Zolensky, M. E. Organic globules in the Tagish Lake meteorite: Remnants of the protosolar disk. *Science* **314**, 1439-1442, doi:10.1126/science.1132175 (2006).

S2 Starkey, N. A., Franchi, I. A. & Lee, M. R. Isotopic diversity in interplanetary dust particles and preservation of extreme ^{16}O-depletion. *Geochimica et Cosmochimica Acta* **142**, 115-131, doi:https://doi.org/10.1016/j.gca.2014.07.011 (2014).

S3 Davidson, J., Busemann, H. & Franchi, I. A. A NanoSIMS and Raman spectroscopic comparison of interplanetary dust particles from comet Grigg-Skjellerup and non-Grigg Skjellerup collections. *Meteoritics & Planetary Science* **47**, 1748-1771, doi:10.1111/maps.12010 (2012).

S4 Messenger, S. Identification of molecular-cloud material in interplanetary dust particles. *Nature* **404**, 968-971 (2000).

S5 Matrajt, G., Messenger, S., Brownlee, D. & Joswiak, D. Diverse forms of primordial organic matter identified in interplanetary dust particles. *Meteoritics & Planetary Science* **47**, 525-549, doi:doi:10.1111/j.1945-5100.2011.01310.x (2012).
